# The role of FTO in m6A RNA methylation and immune regulation in *Staphylococcus aureus* infection-related osteomyelitis

**DOI:** 10.3389/fmicb.2025.1526475

**Published:** 2025-02-06

**Authors:** Sijing Liu, Kai Li, Changhai Long, Mingwu Lao, Biao Ma, Changquan Liu, Haoyuan He, Chunjiang Wang, Wangzhu Chen, Bin Yu

**Affiliations:** ^1^Division of Orthopaedics and Traumatology, Department of Orthopaedics, Nanfang Hospital, Southern Medical University, Guangzhou, China; ^2^Department of Orthopaedic Center, The Second Hospital Affiliated of Guangdong Medical University, Zhanjiang, China; ^3^Department of Orthopaedic Center, Maoming Hospital of Guangzhou University of Chinese Medicine, Maoming, China

**Keywords:** *Staphylococcus aureus*, m6A, FTO, infection, GSEA

## Abstract

**Background:**

Regulators of n6-methyladenosine (m6A) RNA modification play important roles in many diseases; however, their involvement in *Staphylococcus aureus* (*S. aureus*)-related osteomyelitis remains inadequately explored. Therefore, this study aims to investigate the role of m6A in *S. aureus* infection-related osteomyelitis and elucidate its underlying mechanisms.

**Methods:**

We downloaded the *S. aureus* infection-related osteomyelitis infection dataset GSE30119 from the Gene Expression Omnibus database. Initially, we constructed a diagnostic model based on m6A genes and predicted the hub node miRNAs and transcription factors by constructing a protein–protein interaction network. Subsequently, a prognostic model was built using LASSO regression, the receiver operating characteristic curve of the model was plotted, and the predictive performance of the diagnostic model was validated. Further, unsupervised clustering analysis, gene set enrichment analysis (GSEA), and gene set variation analysis (GSVA) were employed to assess immune cell infiltration. Additionally, we validated the expression of fat mass and obesity-associated protein (FTO) in *S. aureus*-infected Raw264.7 macrophages using qPCR and western blotting. Moreover, we conducted si-FTO experiments on mouse Raw264.7 macrophages to investigate the anti-inflammatory regulatory role of si-FTO during *S. aureus* infection.

**Results:**

We identified 19 co-expressed genes closely related to FTO were identified, along with 206 related transcription factor regulatory genes and 589 miRNAs. Enrichment analyses suggested that these genes were involved in pathways related to the proliferation and oxidation of various immune cells, cellular senescence, and various tumors and immune cells, as well as cell cycle-related functions. GSEA revealed that PD-1, TH1, TH2, CTLA4, and other pathways were significantly enriched in patients with high FTO expression. GSVA indicated that the differentially enriched pathways were related to included amino acid metabolism, immunity, and infection. Correlation analysis of immune infiltration revealed that monocytes, M2 macrophages, resting mast cells, and neutrophils were present in normal and diseased samples. Differences in expression were observed between the groups. The western blotting and qPCR analyses confirmed that the protein expression of FTO was reduced in macrophages after infection with *S. aureus*, consistent with the observed changes in mRNA expression. Furthermore, we validated that FTO may influence the regulation of inflammation through the FoxO1/NF-kB pathway.

**Conclusion:**

The m6A RNA methylation regulator FTO may serve as a potential diagnostic marker and therapeutic target, involved in the pathogenesis of *S. aureus* infection-related osteomyelitis. This finding provides new insights into the relationship between FTO-mediated m6A RNA methylation and osteomyelitis.

## 1 Introduction

Osteomyelitis is a disease caused by microbial pathogens that infect bones, leading to inflammatory reactions and bone destruction. Severe cases can result in lifelong disability (Hatzenbuehler and Pulling, 2021). Among the causative agents, *Staphylococcus aureus* (*S. aureus*) is the predominant pathogen (Pimentel de Araujo et al., 2021). Despite advances in diagnostic methods and clinical management, the disease continues to pose significant challenges, particularly in pediatric and adolescent populations (Bouiller and David, 2023). Currently, direct sampling from wounds for culture and antibiotic sensitivity testing is crucial for targeted treatment. Research has suggested that inflammatory cytokines, *S. aureus*-specific antibodies, procalcitonin, and iron death-related markers can be used for the early diagnosis of osteomyelitis (Zhang et al., 2023; Isogai et al., 2020; Shi et al., 2023). However, the specificity of detecting infection-induced osteomyelitis and the effectiveness of treatment are suboptimal, making early detection, diagnosis, and treatment the primary focuses of research.

The role of N6-methyladenosine (m6A) methylation in autoimmune diseases, inflammation, and cancer has gained significant attention recently (Wu et al., 2018). m6A methylation is a common post-transcriptional RNA modification involving enzymes such as m6A methyltransferases (writers), demethylases (erasers), and m6A RNA-binding proteins (readers) (Jia et al., 2008). Fat mass and obesity-associated protein (FTO), a key demethylase, is a promising biological target because of its role in mRNA modification (Gerken et al., 2007). It regulates cellular RNA m6A levels by removing methyl groups from single-stranded (ss) DNA and ssRNA. Recent studies show that during pathogen-induced sepsis, FTO modulates the formation of NLRP3 inflammasomes via the FoxO1/NF-κB pathway in macrophages (Luo et al., 2021). In addition, the METTL3/m6A/miR-193a/BCL2L2 axis is involved in the regulation of myocardial apoptosis and inflammation (Liang et al., 2023). IGF2BP3 also plays a role in the inflammatory state of synovial macrophages in osteoarthritis (Lu et al., 2023). Although the significance of FTO and other m6A-related genes in inflammatory diseases is known, research on their roles in *S. aureus* infection-related osteomyelitis remains limited.

To address this gap, our study delves into the role of FTO and m6A RNA methylation in the pathogenesis of *S. aureus* infection-related osteomyelitis. By leveraging transcriptome-wide differential expression analyses and functional enrichment studies, we identify critical m6A regulators and unravel their associated molecular pathways. Particular attention is given to the expression dynamics of FTO and its regulatory networks, focusing on their influence on immune responses. Additionally, we investigate the interplay between FTO expression and immune cell infiltration to elucidate its mechanistic contributions to immune modulation and disease progression. These findings provide novel insights into the molecular underpinnings of *S. aureus* infection-related osteomyelitis and inform the development of more precise diagnostic and therapeutic strategies.

## 2 Materials and methods

### 2.1 Data and preprocessing

**Figure 1:**
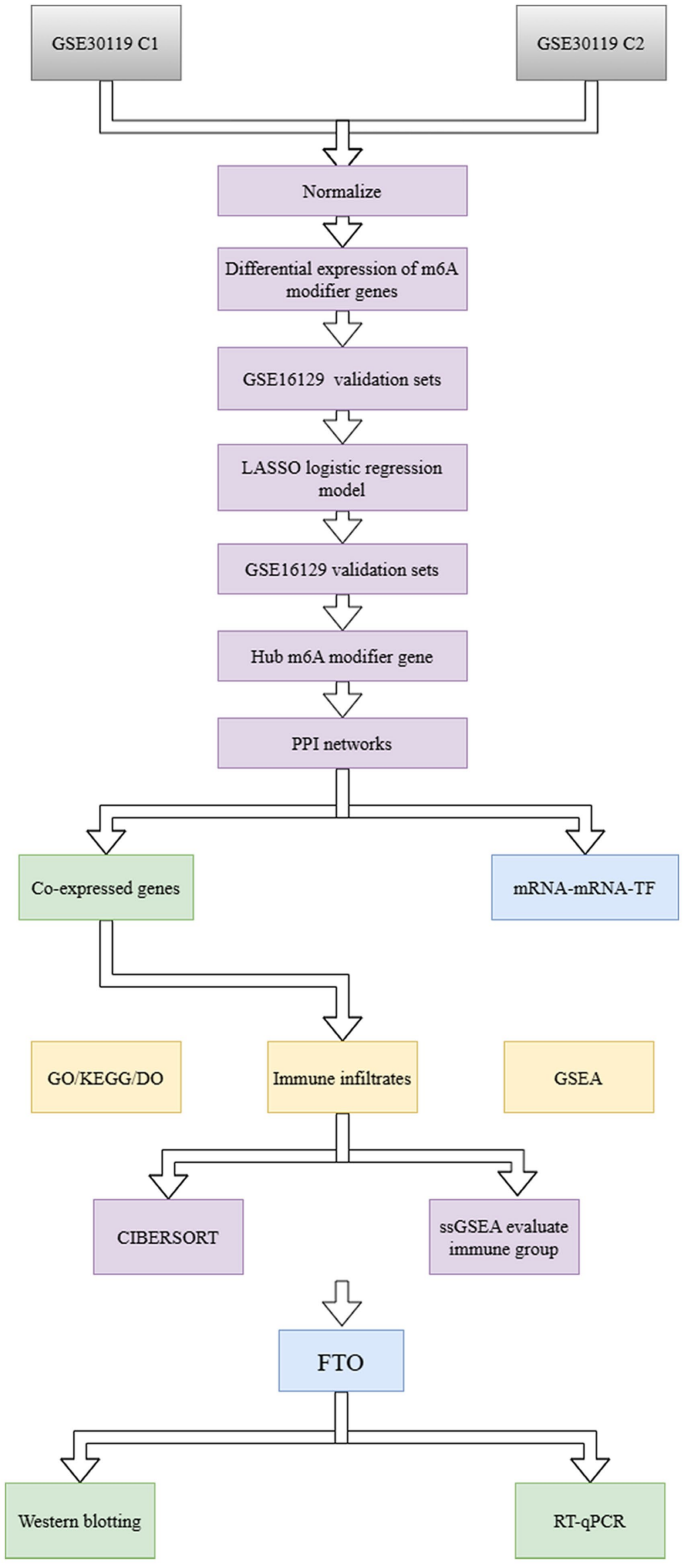
Workflow of the present study.

We downloaded the *S. aureus* infection-related osteomyelitis chip data and the corresponding clinical information for the *S. aureus* infection-related osteomyelitis dataset GSE30119 from the Gene Expression Omnibus database (Toufiq et al., 2000). The sample source was *Homo sapiens*, and the sequencing platform used was GPL6947 Illumina HumanHT-12 V3.0. The dataset included two experimental sets (C1 and C2) representing 44 normal samples and 99 samples from patients with *S. aureus* infections. We included C1 as a training set with 22 normal samples and 40 patients with *S. aureus* infection and C2 as a validation set with 22 normal samples and 59 patients with *S. aureus* infection. In order to validate the model, we collected the bone marrow samples of *S. aureus* infection GSE16129 as the validation set. The R package “sva” was used to correct the batch effect between different datasets and perform log2 standardization (Leek et al., 2012). The expression distributions before and after standardization and batch correction were visualized using a box diagram (Figure 1).

### 2.2 Differentially expressed genes

To analyze the effect of m6A-related genes on disease, the R package “limma” was used to perform differential gene analysis on normal and diseased samples in the dataset (Ritchie et al., 2015). The volcano map showed a log2fold change (logFC) absolute value >2 and Padj <0.05, which were set as the differential expression results, and the heatmap showed the top 50 differentially expressed genes between normal samples and disease samples. Furthermore, we conducted differential analysis specifically focusing on the FTO cluster grouping.

### 2.3 Panorama construction of m6A related genes

To analyze the expression of m6A-related genes in all samples, we first obtained m6A-related genes from the literature (Shi et al., 2019; Chen et al., 2019; Xu et al., 2020; Du et al., 2019), including 11 writer genes, 23 reader genes, and 3 eraser genes, totaling 37 genes. We then intersected the existing expression profiles for 27 genes. First, we used the R package “heatmap” to draw the expression heat map of the genes in all samples, and then we used the “ggpubr” package to draw grouped boxplots based on the normal and patient samples. The Wilcoxon rank sum test method was used for statistical significance between groups, and *p* < 0.05 was considered statistically significant. The “RCircos” package was used to draw a location map of the 27 genes on the chromosome (Zhang et al., 2013). Chromosomal data were provided by the R package, and the location information of the genes on the chromosome was downloaded from the Ensembl database (Yates et al., 2000).

### 2.4 Correlation analysis between writer and eraser genes

To further analyze the correlation between writer and eraser gene expression in all patients, Pearson’s correlation was calculated between the two genes. The absolute value of the correlation coefficient was greater than 0.5 and the *p* value was less than 0.05. We used the R package “ggplot2” to draw a scatterplot of the correlations between gene pairs that met the requirements and fit the correlation curve and used the “ggExtra” package to draw a histogram of the edges of the graph.

### 2.5 Diagnostic model construction based on m6A genes

Owing to the important influence of the m6A modification process, there may be different m6A modification states between normal and patient samples; therefore, we constructed two training groups based on m6A-related genes from GSE30119 as diagnostic models. In addition, we utilized GSE16129 as an external validation dataset. Here, we screened all m6A genes via least absolute shrinkage and selection operator (LASSO) regression using the R package “glmnet” and selected the best lambda value. Only genes with non-zero coefficients were retained. The genes used to construct the model, and their corresponding coefficients were displayed in the form of forest plots using the R package “forestplot.” Subsequently, to reveal the common effect of m6A gene expression on the diagnostic performance of the model, the R package “rms” was used to construct a logistic regression model of the m6A genes with the most significant weights in the previous LASSO model and visualized using a nomogram. To verify the predictive power of the diagnostic model, the R package “pROC” was used to draw single-gene receiver operating characteristic (ROC) curves and calculate the area under the curve (AUC) (Robin et al., 2011). An internal dataset and decision curve analysis (DCA) were used to illustrate the validity of the nomogram; the curves were drawn using the “ggDCA” R package.

### 2.6 Construction of a protein–protein interaction network

The expression of different genes, especially those that regulate the same biological processes, is interrelated. To reveal the relationship between m6A-related genes, a PPI network was constructed based on m6A-related genes using the STRING database with the above genes as inputs and the confidence threshold at the default value of 0.4 (Von Mering et al., 2003). Subsequently, the PPI network was exported and further analyzed using Cytoscape software (Shannon et al., 2003). The network attributes of each node were calculated, and the plug-in cytoHubba was used to mine the hub nodes based on their degree (Chin et al., 2014). The 10 nodes with a degree of TOP10 were defined as hub nodes. These nodes exhibited a high level of connection with other nodes; therefore, they may play an extremely important role in the regulation of the entire biological process and warrant further study. Therefore, we conducted further prediction research on the 10 hub nodes based on the miRNet database to predict the miRNAs and transcription factors of the hub nodes (Chang et al., 2020). The predicted results were processed and plotted using Cytoscape software.

### 2.7 Construction of a diagnostic model based on hub genes

All hub genes were screened using LASSO regression, and a LASSO model was constructed and displayed as a forest graph. To verify the predictive power of the diagnostic model, the R package “pROC” was used to draw the ROC curve of the model and calculate the AUC.

### 2.8 Unsupervised clustering of samples

Because of the pervasive heterogeneity among samples, unsupervised clustering of samples based on m6A regulators was applied to resolve heterogeneity and reclassify samples. Different m6A modification patterns were identified based on the expression of m6A regulators. Clustering was performed using the R package “ConsensusClusterPlus,” and the number of clusters was estimated (Wilkerson and Hayes, 2010). The basic principle of consensus clustering assumes that samples extracted from different subclasses of the original dataset constitute a new dataset, and that different samples from the same subclass are extracted and clustered on the new dataset. Accordingly, both the numbers of clusters and samples within the class should be similar to those of the original dataset. Therefore, the more stable the resulting cluster is with respect to the sampling variation, the more representative the cluster is of a true subclass structure. The resampling method could disrupt the original dataset, so clustering analysis was performed on each resampling sample, and then the results of multiple clustering analyses were comprehensively evaluated to determine the consensus.

### 2.9 Functional enrichment analysis of differentially expressed genes in *Staphylococcus aureus* infection

To reveal the biological differences between the two sample groups, we conducted Gene Set Enrichment Analysis (GSEA) and displayed the results using volcano and heat maps. Significant differentially expressed genes (DEGs) were defined as having a corrected *p* value <0.05 and |log2FC| > 0.5.

We performed Gene Ontology (GO) enrichment analysis on significant DEGs to annotate their functions, focusing on three categories: biological process, molecular function, and cellular component (Ashburner et al., 2000). The Kyoto Encyclopedia of Genes and Genomes (KEGG) database provides valuable information on genomes and pathways (Kanehisa and Goto, 2000). The R package GOPlot and KEGGPlot were used to annotate the GO and KEGG functions of all significant DEGs to identify the enriched biological processes (Yu et al., 2012). GSEA helps determine statistical differences in predefined gene sets between two biological states and assesses changes in pathway activity (Subramanian et al., 2005). We downloaded reference gene sets “c5.go.v7.4.Entrez.GMT” and “c2.cp.kegg.v7.4.Entrez.GMT” from the MSigDB database and performed GSEA using “clusterProfiler,” with *p* < 0.05 as the significance criterion (Liberzon et al., 2015). Gene Set Variation Analysis (GSVA) is a non-parametric method that converts gene expression matrices into gene set expression matrices to evaluate pathway enrichment across samples (Hänzelmann et al., 2023). To examine biological process variation between the two groups, we calculated enrichment scores for each pathway from the “c2.cp.kegg.v7.4.Entrez.GMT” dataset and used the “limma” package to identify significantly differential pathways. GSVA results were visualized using “pheatmap” considering p < 0.05 as statistically significant.

### 2.10 Immune infiltration analysis

To further explore the similarities and differences in immune cell infiltration levels between the two groups of samples, the “GSVA” package was used following the single-sample GSEA (ssGSEA) method. The marker genes of the 28 immune cells were obtained from the literature and used as the background gene set for ssGSEA of each sample (Charoentong et al., 2017). The infiltration of all immune cells was visualized using box plots. Simultaneously, the R package “corrplot” was used to draw a correlation map between the immune cells for the two groups of samples to reveal the similarities and differences in the degree of correlation of immune cells in different cancer states. In addition, to directly view the correlation between the hub genes and the level of immune cell infiltration, a correlation scatter plot was drawn for the gene–immune cell pairs with significant correlations, and a correlation curve was fitted.

To maximize the accuracy of the results, the R package “CIBERSORT” was used to evaluate the infiltration level of immune cells (Steen et al., 2020), and the content of 22 immune cells in each sample was calculated based on the LM22 background gene set provided by CIBERSORT to reflect the infiltration level. CIBERSORT is based on the principle of linear support vector regression to deconvolute the transcriptome expression matrix and estimate the composition and abundance of immune cells in mixed cells. The results were displayed using heat maps and stacked bar charts drawn using the R package “ggplot2.” For gene–immune cell pairs with significant correlations, we drew a correlation scatterplot and fit a correlation curve. Samples with *p* < 0.05 were included to obtain the immune cell infiltration matrix.

### 2.11 Correlation analysis of hub genes

In order to analyze the correlation between these hub genes, a correlation heat map was drawn using the R package “corrplot.” In addition, in order to further study the correlation of these genes with endoplasmic reticulum stress and mitophagy process, relevant genes were retrieved from the GeneCards database with the keywords “endoplasmic reticulum stress” and “mitophagy,” and then the correlation between hub genes and these genes was calculated and visualized in the form of a bubble chart (Stelzer et al., 2016). For the most significant hub gene pairs, a correlation scatter plot was drawn and a correlation curve was fitted.

### 2.12 Isolation of, and infection with, *Staphylococcus aureus*

*Staphylococcus aureus*, a pathogen causing asteomyelitis, preserved and provided by the laboratory (Guangdong Provincial Key Laboratory of Bone and Cartilage Regeneration Medicine, Nanfang Hospital, Southern Medical University Guangzhou, Guangdong, China). Prior to conducting the infection experiments, *S. aureus* was added to 10 mL of fresh tryptic soy broth and incubated overnight at 37°C with agitation at 120 rpm. After centrifugation, the bacteria were washed three times with phosphate-buffered saline (PBS) and resuspended in PBS. The concentration of *S. aureus* was adjusted to an optical density of 0.5 at 600 nm, which is approximately equivalent to 1 × 10^8^ colony-forming units per milliliter (CFU/mL), to ensure consistent inoculum densities (Lu et al., 2023). The resulting bacterial suspension was appropriately diluted for infection experiments in a RAW 264.7 macrophage cell line (Procell, Wuhan, China). Cells were seeded at a density of 1 × 10^6^ cells/well in a 6-well plate and cultured in Dulbecco’s modified Eagle’s medium (PM150210, Procell) supplemented with 10% fetal bovine serum (FBS; 164,210–50, Procell) and 1% penicillin–streptomycin (PB180120, Procell). To evaluate the gene expression response of macrophages to *S. aureus* infection, cells were infected with *S. aureus* at doses of 100, 10, and 1× the multiplicity of infection (MOI). After 1 h of infection, cells were treated with 20 μg/mL gentamicin (215–778-9, Sigma-Aldrich, St. Louis, MO, United States) for 30 min to kill any remaining extracellular *S. aureus*, thus eliminating extracellular bacteria. After washing thrice with PBS, the cells were incubated in fresh culture medium containing 10% FBS for an additional 24 h. Subsequently, the RNA was collected for mRNA expression analysis.

### 2.13 Total RNA extraction and real-time quantitative PCR

RAW 264.7 cells were lysed using TRIzol reagent (TaKaRa Bio, Kusatsu, Japan) to isolate RNA. Subsequently, the RNA was reverse transcribed into cDNA using the Uni All-in-One First-Strand cDNA Synthesis SuperMix for qPCR kit (TransGen Biotech, Beijing, China). qPCR was performed using CFX96 (Bio-Rad, Hercules, CA, United States) and RealStar Power SYBR qPCR Mix (GenStar, Beijing, China). This method allows for the detection and quantification of gene expression levels in cDNA samples. To ensure accurate normalization and comparison of gene expression data, the relative expression of the target genes was normalized to that of the reference gene, *GAPDH*. Data analysis and quantification were performed using the 2^–ΔΔCt^ method, which calculates the fold change in gene expression relative to a control sample. The PCR primers were 5′-GACACTTGGCTTCCTTACCTG-3′ (FTO forward) and 5′-CTCACCACGTCCCGAAACAA-3′ (FTO reverse); 5′-AGGTCGGTGTGAACGGATTTG-3′(GAPDH forward) and 5′-GGGGTCGTTGATGGCAACA-3′ (GAPDH reverse).

### 2.14 Western blotting

To collect RAW 264.7 cells infected with *S. aureus*, Cell Lysis Buffer for Western or IP (P0013, Beyotime, Shanghai, China) supplemented with a protease inhibitor cocktail (ST506-2, Beyotime), was used for protein extraction and purification. Equal amounts of protein were separated by SDS-PAGE on a 10% polyacrylamide gel and then transferred onto a PVDF membrane. The membrane was blocked with skim milk at room temperature for 1 h and washed thrice with TBST. Subsequently, the membranes were incubated overnight with primary antibodies against FTO (1:1000; 41,548, Signalway Antibody LLC, College Park, MD, United States), *β*-actin (1:1000; 52,901, Signalway Antibody LLC), Anti-IL1B (Signalway Antibody LLC, dilution 1:1000), Anti-IL6 (Signalway Antibody LLC, dilution 1:1000), Anti-NFKB (Signalway Antibody LLC, dilution 1:1000), Anti-p-NFKB (Signalway Antibody LLC, dilution 1:1000), and Anti-FOXO1 (Signalway Antibody LLC, dilution 1:1000). The following day, the membrane was washed thrice with TBST and incubated with horseradish peroxidase-conjugated goat anti-rabbit IgG secondary antibody (1:1000; L3012, Signalway Antibody LLC). Finally, images were captured using GelView 6000Plus (Biotend, Guangzhou, China).

### 2.15 Statistical analysis

All data processing and analysis was performed using Excel (Microsoft, Redmond, WA, United States) and R software (version 4.0.2). For comparisons of two groups of continuous variables, the statistical significance of normally distributed variables was estimated using an independent Student’s t-test, and the differences between non-normally distributed variables were analyzed using the Mann–Whitney U test (i.e., Wilcoxon rank sum test). The chi-squares test or Fisher’s exact test was used to compare and analyze the statistical significance of categorical variables between the two groups. The Kruskal–Wallis test was used to compare two or more groups, and the Wilcoxon test was used to compare two groups. ROC curves were drawn using the pROC package in R, and the AUC was calculated to assess the accuracy of the risk score in estimating prognosis. All statistical *p* values were two-sided, and *p* < 0.05 considered statistically significant. Two-tailed p values <0.05 was considered statistically significant.

## 3 Results

### 3.1 Data preprocessing

**Figure 2:**
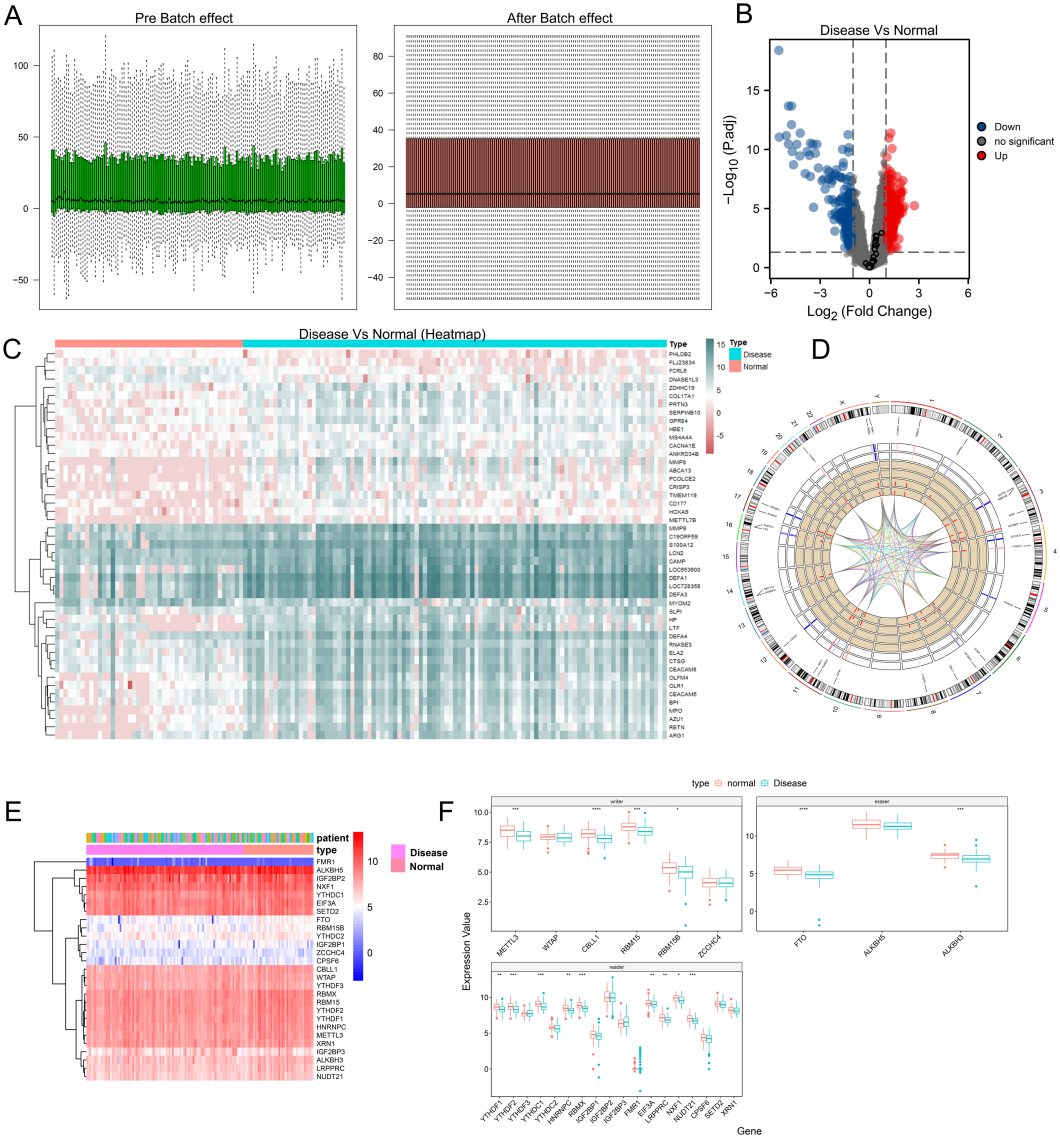
Comparison and analysis between different experimental groups. **(A)** Effect of batch variation; the left panel shows data distribution before batch effect, while the right panel shows data distribution after batch effect. **(B)** Volcano plot illustrating differences between disease and normal groups; blue dots represent downregulated genes, red dots indicate upregulated genes, and black dots represent genes with no significant differences. **(C)** Heatmap displaying expression patterns of samples from disease and normal groups, clustered by similarity. **(D)** Circular genomic plot showing the distribution of m6A-related genes across different chromosomes. **(E)** Heatmap further illustrating the expression levels of m6A-related genes in patient samples, with colors ranging from red to blue indicating varying expression levels. **(F)** Boxplot comparing the expression levels of m6A-related genes between disease and normal groups, highlighting significant differences (* *p* < 0.05, ** *p* < 0.01, *** *p* < 0.001, **** *p* < 0.0001).

We first examined the gene expression distribution of the original expression profile of the GSE30119 dataset before and after batch effect correction. The samples showed a serious batch effect when integrated directly, and samples from different sources showed significantly different expression distribution characteristics. After batch effect correction and log normalization, the expression distribution of all samples tended to be consistent, improving the accuracy and robustness of the downstream analysis (Figure 2A). The data spectrum expression distribution of the GSE30119 dataset before and after standardization correction is shown in the box diagram. The heatmap and volcano map show the difference in expression between normal and diseased samples in the dataset GSE30119 (Figures 2B,C).

### 3.2 Panorama of m6A genes

The expression heterogeneity of all m6A genes in normal and diseased samples was visualized using a heat map and a grouping box diagram (Figures 2E,F). The results showed that among the three m6A gene types, the difference between the readers in the two groups was more obvious than that between writers and erasers. To construct a panorama of m6A-related genes in all samples, we examined the localization of these genes on chromosomes and found that some genes were very close to each other, indicating that they were closely related at the genomic level (Figure 2D). The results showed that some genes were very close to each other on the chromosome, indicating that these genes were closely related at the genomic level. To further analyze the relationship between the expression of m6A writer and eraser genes, we calculated the correlation between these genes and obtained the correlation coefficients *R* > 0.5 and *p* < 0.05, indicating positive correlations (Supplementary Figure S1).

### 3.3 m6A gene-based diagnostic model

Since the expression of m6A-regulated genes has important biological significance, we constructed a diagnostic model for *S. aureus* infection based on all m6A genes.

**Figure 3:**
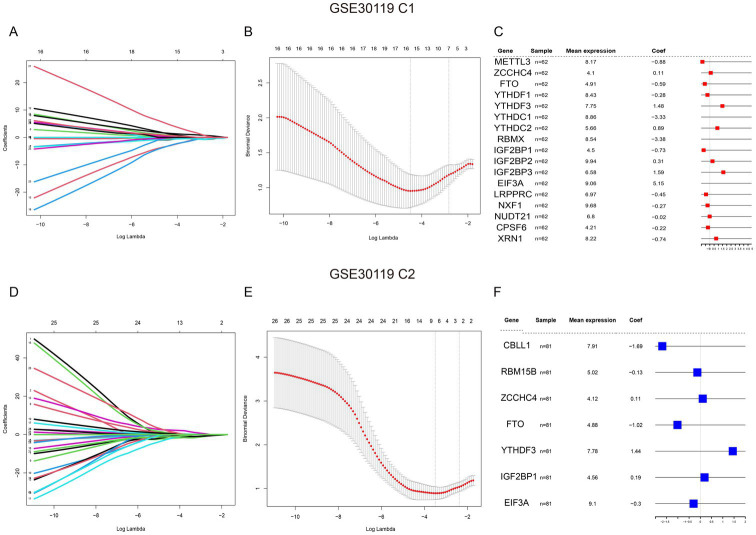
Analysis of gene expression in GSE30119 datasets. **(A)** Coefficient plot for the GSE30119 C1 dataset, displaying the relationship between coefficients and log lambda values for various genes. **(B)** Minimum cross-validated error plot for GSE30119 C1, showing the relationship between log lambda and the distance metric with error bars indicating variability. **(C)** Table of significant genes identified in GSE30119 C1, including mean expression levels and coefficients, with highlights on important genes. **(D)** Coefficient plot for the GSE30119 C2 dataset, similar to (A), showing the coefficients for different genes across log lambda values. **(E)** Minimum cross-validated error plot for GSE30119 C2, demonstrating the relationship between log lambda and the distance metric. **(F)** Table of significant genes identified in GSE30119 C2, detailing mean expression levels and coefficients, highlighting key regulatory genes in the analysis.

First, the 27 m6A genes in the training and validation sets were regressively screened using LASSO, and the best lambda values were obtained for 17 genes in training set C1 (Figures 3A,B) and the 7 genes in validation set C2 (Figures 3D,E). Subsequently, forest maps were used to visualize the effects of these m6A gene-containing diagnostic models in the training (Figure 3C) and validation sets (Figure 3F).

Subsequently, to verify the accuracy of the model, we created nomograms and found that in the GSE30119 training set C1, the patient prediction risk score correlated with the disease risk of patients with *S. aureus* infection, highlighting the accuracy of the model (Supplementary Figure S2A). The predictive performance of the model was further validated using recall curves and decision curve analysis (DCA), which confirmed that the model exhibited superior predictive performance and robustness (Supplementary Figures S2C,E). Additionally, the nomogram model, recall curves, and DCA demonstrated the same efficacy as observed in the C1 validation set (Supplementary Figures S2B,D,F).

### 3.4 Differential expression of key m6A regulators and diagnostic efficacy verification

**Figure 4:**
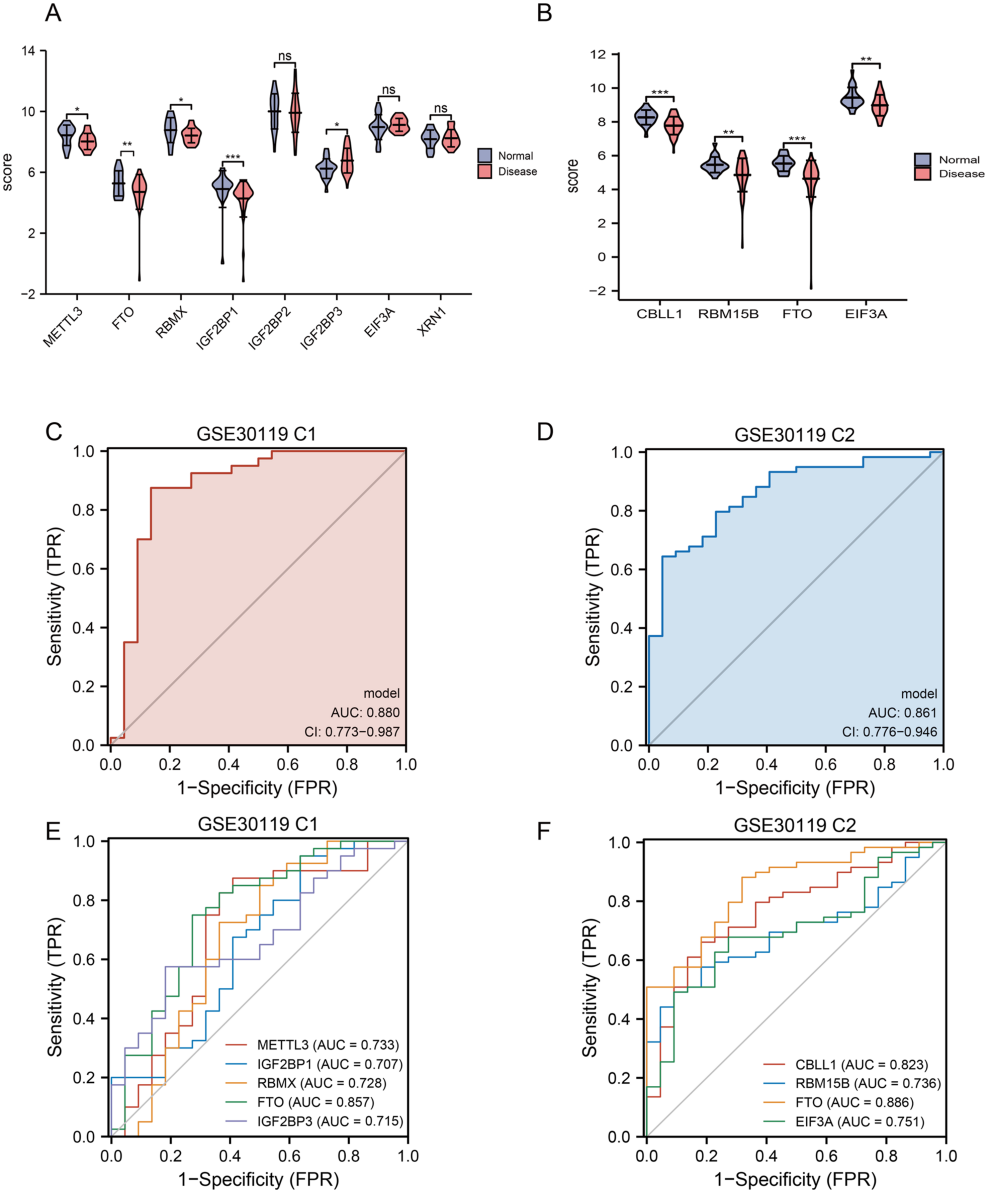
Gene expression and ROC analysis in GSE30119 datasets. **(A)** Violin plot showing the expression scores of selected genes in normal and disease groups, with asterisks indicating significant differences (**p < 0.05; **p < 0.01; ****p < 0.0001) and “ns” indicating no significant difference. **(B)** Boxplot comparing the expression scores of key genes (CBLL1, RBM15B, FTO, and EIF3A) between normal and disease groups, with significant differences highlighted. **(C)** ROC curve for GSE30119 C1, displaying sensitivity versus 1-specificity, with an AUC of 0.880 and confidence interval (CI) indicating model performance. **(D)** ROC curve for GSE30119 C2, illustrating a sensitivity of 0.861 with an AUC of 0.861 and confidence interval. **(E)** ROC comparison for multiple genes in GSE30119 C1, showing AUC values for each gene, with METTL3, IGF2BP1, RBM5, FTO, and IGF2BP3 indicated. **(F)** ROC comparison for multiple genes in GSE30119 C2, presenting AUC values for CBLL1, RBM15B, FTO, and EIF3A, highlighting their diagnostic potential.

To further identify the key m6A regulators for diagnosing patients with the disease, we compared the factors with diagnostic significance in the two sets and found that only FTO was differentially expressed (Figures 4A,B). The AUC for FTO as a diagnostic marker for *S. aureus* infection was 0.857 in the training set and 0.886 in the validation set (Figures 4E,F). Moreover, the difference in FTO expression between different disease groups was significant, suggesting that FTO has high diagnostic value. Combined with the above forest diagram, it was confirmed that FTO is a low-risk gene for *S. aureus* infection.

To validate and analyze whether the diagnostic models based on the LASSO algorithm for C1 and C2 could independently distinguish between diseases, we plotted ROC curves and calculated the AUC. The AUC for C1 was 0.880, and for C2, it was 0.861, indicating good diagnostic efficacy (Figures 4C,D). Furthermore, to enhance the validation of diagnostic performance, we selected the GSE16129 dataset related to *S. aureus* infection osteomyelitis for external validation. LASSO regression, forest plots, and nomograms all confirmed that FTO demonstrated diagnostic efficacy (Supplementary Figures S3A–D). Additionally, calibration curves and decision curve analysis (DCA) further supported these findings (Supplementary Figures S3E,F).

### 3.5 Molecular typing analysis of the key diagnostic marker FTO

**Figure 5:**
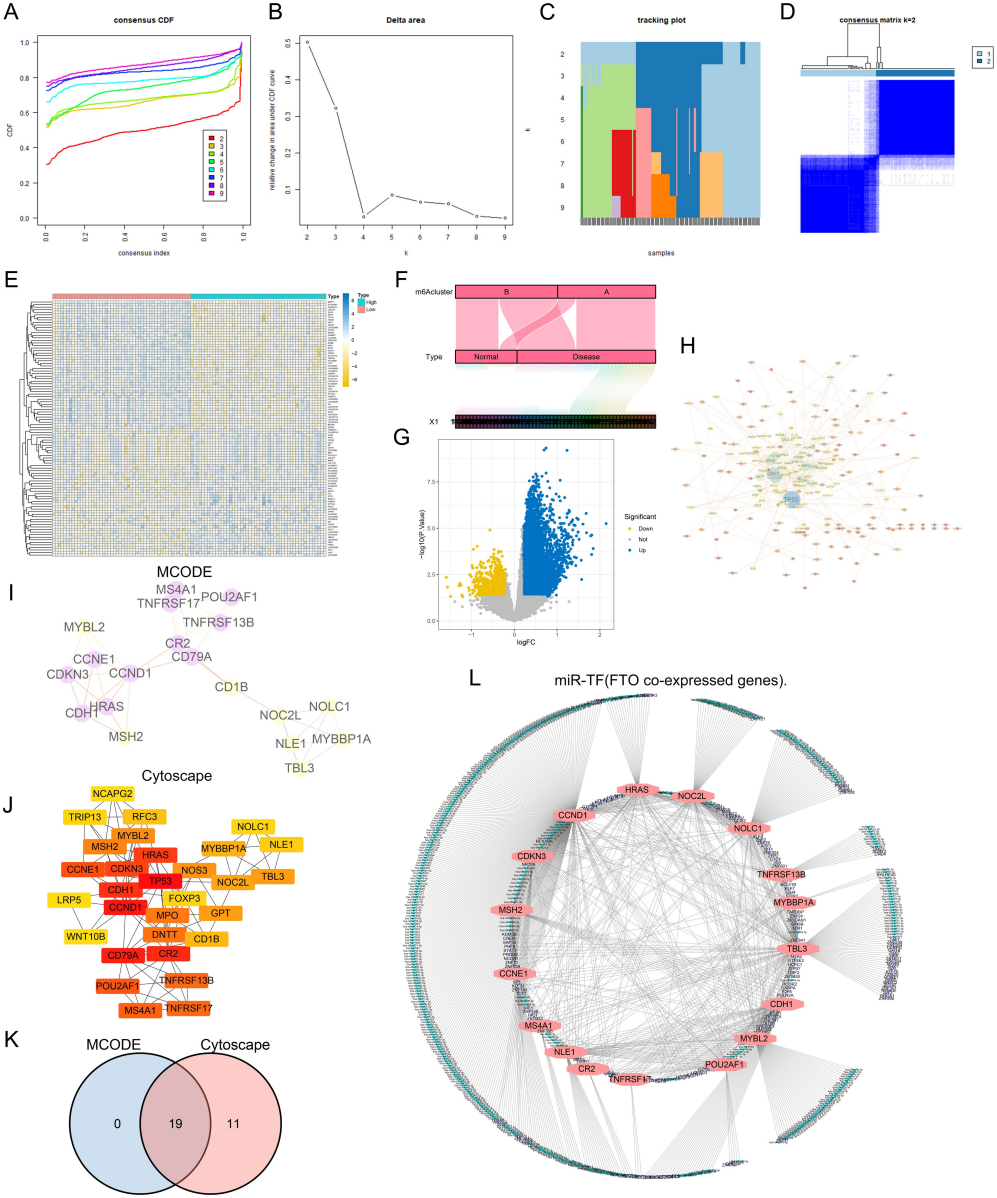
Analysis and visualization of gene co-expression and clustering about FTO. **(A)** Consensus clustering CDF illustrating the stability of different cluster solutions, with each line representing a different cluster number. **(B)** Delta area plot showing the change in area under the CDF curve, indicating optimal cluster selection. **(C)** Tracking plot displaying the assignment of samples to clusters across different consensus clustering iterations. **(D)** Heatmap of consensus matrix, visualizing the clustering results and similarity between samples (m6A Cluster). **(E)** Hierarchical clustering heatmap of gene expression, showing distinct patterns between different m6A Cluster type. **(F)** Alluvial diagram representing the distribution of sample types (Normal vs. Disease) across identified clusters. **(G)** Volcano plot highlighting significant genes, with blue indicating downregulated and yellow indicating upregulated genes. **(H)** Network visualization showing interactions between significant genes, with nodes representing genes and edges representing co-expression relationships. **(I)** MCODE analysis identifying densely connected gene modules within the co-expression network. **(J)** Cytoscape hub representation of identified gene modules, with highlighted nodes indicating key genes. **(K)** Venn diagram comparing gene overlap between MCODE and Cytoscape analyses, indicating shared and unique genes. **(L)** Network diagram of miR-TF (FTO co-expressed genes), illustrating the relationships among identified genes in the regulatory network.

To investigate the role of the key diagnostic marker FTO in disease, we conducted unsupervised consensus clustering on 143 samples from the GSE30119 dataset based on gene expression. The disease samples were clustered according to different m6A regulator subclasses, leading to an optimal k value of 2 (Figures 5A–C), which allowed us to classify the samples into two distinct subtypes (A: *n* = 70; B: *n* = 73, Figure 5D). We then compared the cluster groupings with high- and low-expression FTO levels, visualizing the results in a Sankey diagram (Figure 5F). Notably, Class A predominantly represented the disease samples, suggesting that FTO may play a significant role in differentiating between FTO Cluster A and FTO Cluster B.

### 3.6 PPI network construction of co-expressed genes based on the m6A regulator FTO

To further analyze the influence of FTO on diseases, we analyzed the differences between groups with high and low FTO expression and obtained a total of 645 co-expressed genes that may be subject to the same regulatory processes and reflect similar biological functions (*p* value <0.05 and | log2FC | > 1) (Figure 5G). Additionally, I selected the top 50 genes for visualization in a heatmap (Figure 5E).

To gain a deeper understanding of FTO-related differential genes and their biological significance, we utilized the STRING database was used to construct a protein–protein interaction network of differential genes for the 645 FTO co-expressed genes (Figure 5H). The tightly linked genes of the PPI network module were screened using the MCODE plug-in in CYTOSCAPE (v3.7.2), and the highest confidence interaction score was set to 0.4 and visualized (Figure 5I). Simultaneously, the cytoHubba plug-in was used to screen the Top 30 closely linked genes (Figure 5J). The intersection of the two methods is shown in a Venn diagram, which shows 19 closely related FTO co-expressed genes (Figure 5K). These genes are closely related to FTO expression.

Subsequently, to further understand the interaction of the 19 FTO co-expressed genes at the post-transcriptional stage, we identified 589 miRNAs and 206 transcription factor regulatory genes targeted by them and constructed a network (Figure 5L).

### 3.7 GO/KEGG/GSEA enrichment analysis

**Figure 6:**
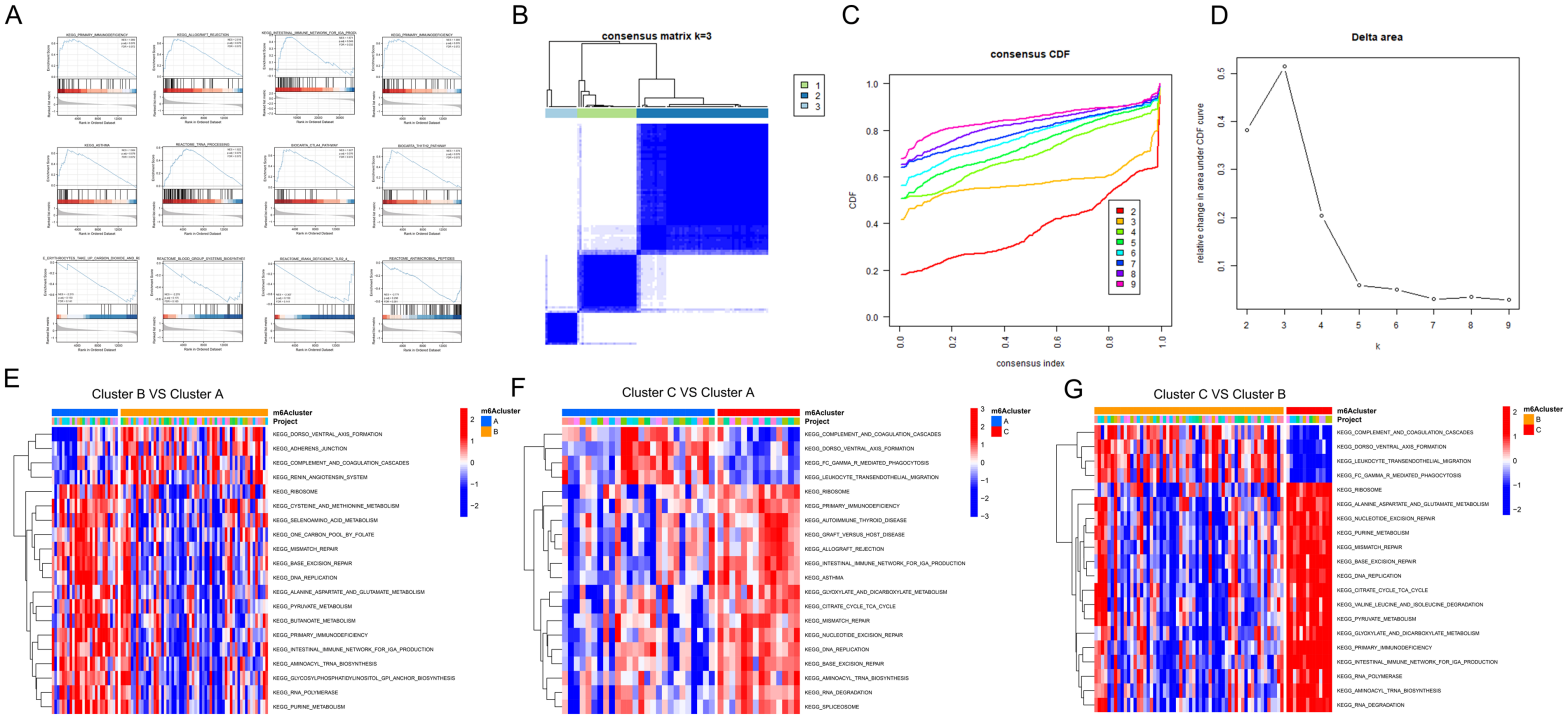
Clustering and pathway analysis of gene expression data about 19 FTO co-ohub genes. **(A)** Visualization of the enrichment scores for various KEGG pathways across different clusters, highlighting the primary pathways associated with each cluster. **(B)** Consensus matrix for k = 3, illustrating the clustering of samples with the dendrogram indicating sample relationships (19 FTO co-ohub genes). **(C)** Consensus cumulative distribution function (CDF) plot showing the stability of cluster assignments as a function of consensus index, with different colors representing varying cluster numbers. **(D)** Delta area plot indicating the relative change in area under the CDF curve, helping determine the optimal number of clusters. **(E)** Heatmap comparing Cluster B versus Cluster A, displaying the expression patterns of selected pathways, with colors indicating the magnitude of expression changes. **(F)** Heatmap comparing Cluster C versus Cluster A, showcasing distinct pathway expressions and their significance in the analysis. **(G)** Heatmap comparing Cluster C versus Cluster B, further illustrating the differential expression of pathways among the clusters.

GO analysis showed enrichment in DNA binding, mismatch repair complex binding, and cell cycle. Function-related genes were enriched in immune cell proliferation, differentiation, activation, and oxidation (Supplementary Figure S4). The KEGG enrichment results indicated that the entries were enriched in various cellular senescence, tumor, and immune cell-related pathways. GSEA enrichment analysis was performed between the groups with high and low FTO expression (c2.all.v7.2.symbols.gmt was used as the background set) (Supplementary Tables S1–S3). The results suggested that the related molecular mechanisms mediated by PD-1, TH1TH2, CTLA4, and other pathways were significantly enriched in patients with high FTO expression (Figure 6A).

### 3.8 Molecular typing analysis of co-expressed hub genes associated with FTO

To further investigate the biological characteristics of FTO expression in diseased tissues, we performed unsupervised consensus clustering using the expression of 19 hub co-expression molecules associated with FTO. Cluster analysis was conducted on disease sample data to classify the samples into different subclasses based on these molecules. After evaluating multiple cluster analyses for consistency, we selected an optimal k value of 3 based on the delta plot, resulting in three distinct subtypes (A: *n* = 26; B: *n* = 58; C: *n* = 14; Figures 6B–D).

Subsequently, we performed Gene Set Variation Analysis (GSVA) between the groups, focusing on comparisons among Cluster C vs. Cluster A, Cluster C vs. Cluster B, and Cluster B vs. Cluster A, using the c2.all.v7.2.symbols.gmt background set. The differentially enriched pathways identified included amino acid metabolism and immune- and infection-related processes (Figures 6E–G).

### 3.9 Evaluation and analysis of immune cell infiltration

**Figure 7:**
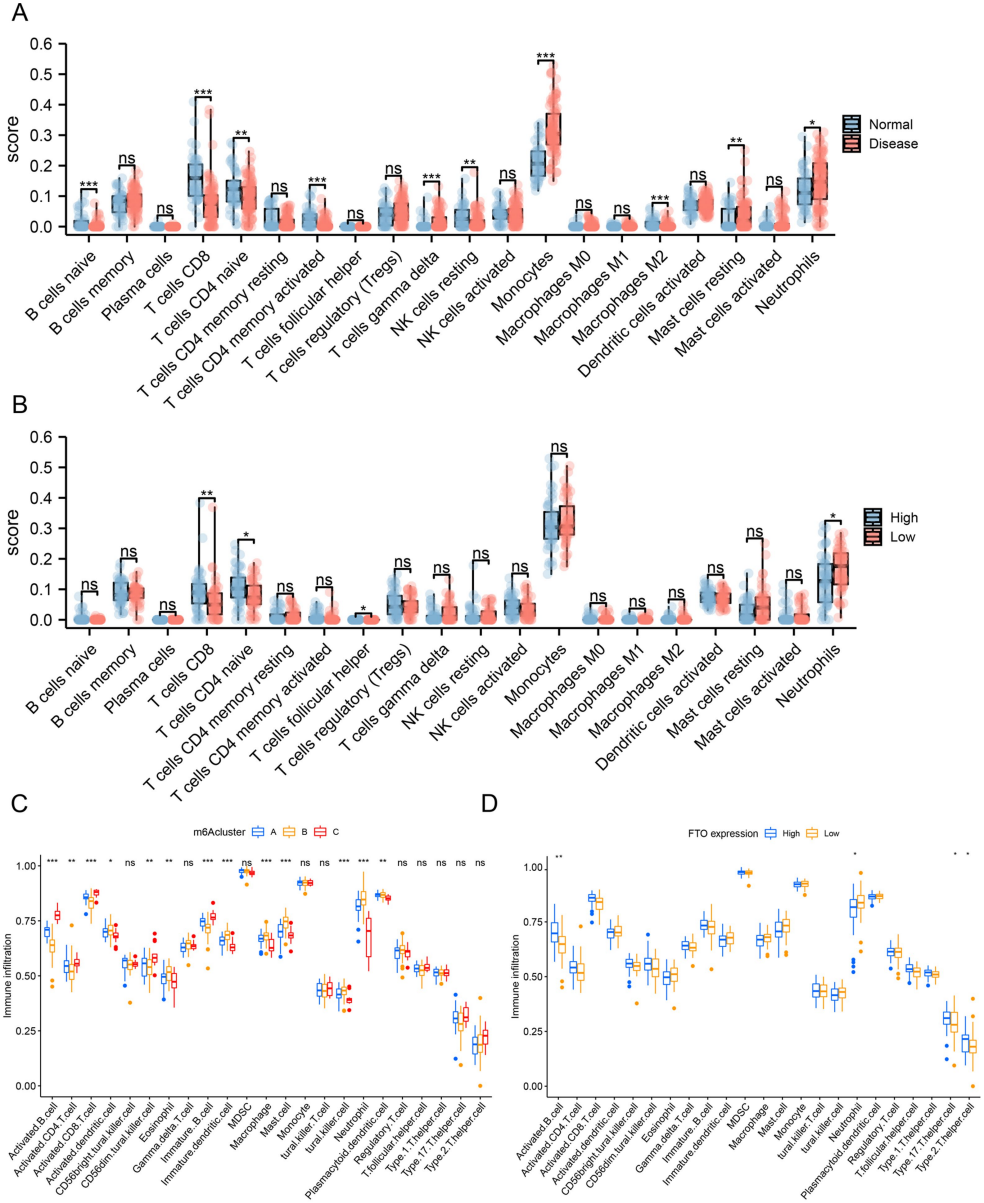
Differential analysis of immune infiltration. **(A)** Box plot from the CIBERSORT algorithm to evaluate the difference in immune cell infiltration between the normal and disease sample groups. **(B)** Box plot from the CIBERSORT algorithm to evaluate the difference in immune cell infiltration between the FTO high and low expression groups. **(C)** ssGSEA algorithm evaluating the differences in immune cell infiltration among the three clusters from the hub co-expression molecular typing of FTO. **(D)** ssGSEA algorithm evaluating the differences in immune cell infiltration between the high and low FTO expression groups.

Immune cell infiltration was assessed using the GSE30119 dataset. The CIBERSORT algorithm is shown in a box chart to evaluate the difference in immune cell infiltration between the normal and disease sample groups (Figure 7A), and the immune infiltration correlation analysis was further performed on the disease-related diagnostic prediction model according to the difference in immune cell expression between the FTO high and low expression groups (Figure 7B). The expression of naïve B cells, CD8+ T cells, naïve CD4+ T cells, activated memory CD4+ T cells, gamma delta T cells, resting natural killer cells, monocytes, M2 macrophages, resting mast cells, and neutrophils differed between the normal and disease sample groups. The number of CD8+ T cells, naïve CD4+ T cells, T cell helper cells, and neutrophils differed between the high and low FTO expression groups.

We then performed ssGSEA to estimate the number of specific infiltrating immune cells and the specific immune response activity, which defined an enrichment score to determine the absolute enrichment of a gene set in each sample in the dataset. We compared the differences in immune cell infiltration among the three clusters from FTO hub co-expression molecular typing (Figure 7C) and between the high and low FTO expression groups (Figure 7D).

### 3.10 Correlation analysis of the key m6A regulator FTO with immune cells in disease samples

To analyze the correlation between FTO expression and immune cells in diseased samples, we drew a scatter plot showing the expression correlation scatter plot between the genes with the strongest correlation (correlation coefficient R > 0.6 and significant sex *p* < 0.01). Spearman correlation analysis was used to determine the correlation between FTO expression, the proportion of immune cells, and immune reactivity. The results showed that FTO expression was closely related to Activated.B.cell (*R* = 0.548), Immature.B.cell (*R* = 0.479), T.follicular.helper.cell (*R* = 0.444), Activated.CD8.T.cell (*R* = 0.437), MDSC (*R* = 0.418), and type.17.T.helper cells (*R* = 0.402), with a positive correlation (Supplementary Figures S5A–F).

### 3.11 Influence of *Staphylococcus aureus* infection on FTO expression in RAW 264.7 macrophages

**Figure 8:**
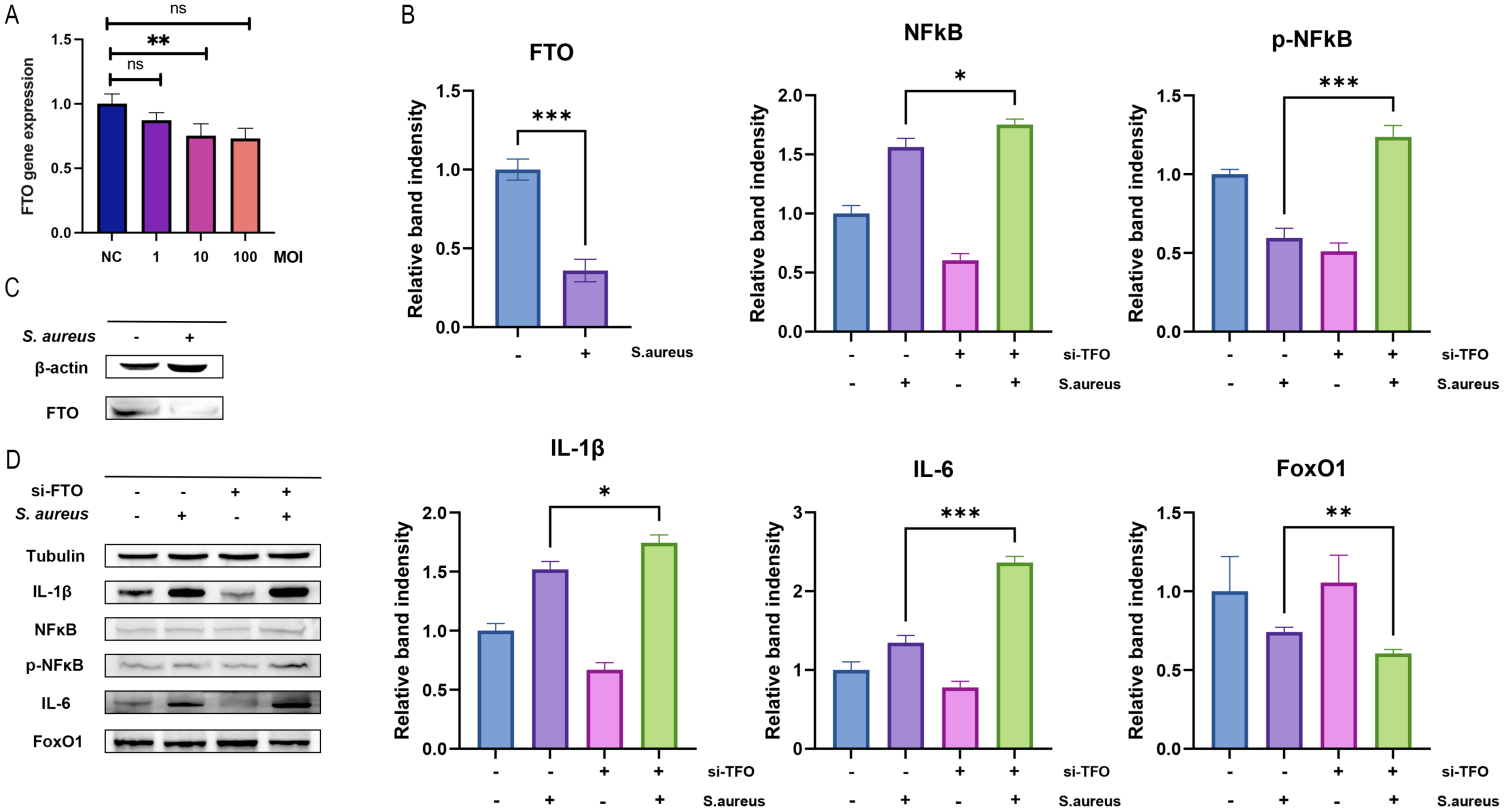
FTO expression and its effects on inflammatory markers in Raw264.7 cells. **(A)** Bar graph showing FTO gene expression levels in different experimental conditions (NC: negative control, 1, 10, 100 MOI), with significant differences indicated (**p < 0.01, ns: not significant). **(B)** Relative band intensity of FTO, NFkB, and phosphorylated NFkB (p-NFkB) protein levels, demonstrating significant changes in response to *S. aureus* infection and si-FTO treatment (*p < 0.05; **p < 0.01; ***p < 0.001). **(C)** Western blot analysis of FTO expression in Raw264.7 cells with and without *S. aureus* infection, with *β*-actin as a loading control. **(D)** Western blot analysis of inflammatory markers (IL-1β, NFkB, p-NFkB, IL-6, FoxO1) in Raw264.7 cells treated with si-FTO and *S. aureus*, with tubulin as a loading control, highlighting significant differences in band intensity for IL-1β and IL-6.

To investigate the effect of *S. aureus* infection on FTO expression in macrophages, RAW 264.7 cells were infected with different MOIs of *S. aureus*. We observed a significant decrease in both the mRNA and protein levels of FTO as the MOI increased. Following infection with *S. aureus* at an MOI of 10, the FTO expression in macrophages was statistically significant (Figure 8A). Furthermore, Western blot experiments confirmed that FTO protein expression was reduced in macrophages after infection with *S. aureus* at an MOI of 10, which was consistent with the observed changes in mRNA expression (Figure 8C). Based on the groups—normal group, *S. aureus*-infected macrophage group, si_Fto group, and *S. aureus*-infected macrophage + si_FTO group, we conducted Western blot analysis to assess the expression of inflammatory proteins and the FOXO1/NFKB signaling pathway proteins. We found that in the *S. aureus*-infected macrophage group and the *S. aureus*-infected macrophage + si_FTO group, IL6 and IL1β inflammatory protein levels increased following si_FTO treatment. Additionally, we detected the expression of p-NFKB, NFKB, and FOXO1 proteins, suggesting that FTO may regulate the macrophage phenotype through the FOXO1/NFKB pathway (Figure 8D). Additionally, we performed statistical analysis on the protein bands using bar charts, which demonstrated significant differences among the groups (Figure 8B).

## 4 Discussion

Up to 75% of osteomyelitis cases are caused by *S. aureus*, a pathogen that is notoriously difficult to diagnose in the early stages due to its nonspecific symptoms. Moreover, the increasing prevalence of antibiotic resistance complicates treatment, making management of these infections a significant challenge (Chen et al., 2019; Maiti and Jiranek, 2014). Consequently, *S. aureus* bone infections have attracted considerable research attention. Understanding the interaction between microbial pathogens and bone tissue, as well as the molecular mechanisms governing phagocytic function in immune responses, is critical for developing novel treatments that mitigate antibiotic resistance and prevent chronic infections.

Recent research on m6A RNA methylation has revealed its pivotal role in RNA metabolism, influencing splicing, expression, and translation (Desrosiers et al., 1974; Gilbert et al., 2016). Among the various m6A regulators, FTO stands out due to its potential as a diagnostic marker in *S. aureus* infections. Our study demonstrated that FTO expression was significantly upregulated in *S. aureus* infections, with AUC values of 0.857 in the training set and 0.886 in the validation set, highlighting FTO’s strong diagnostic value. Furthermore, FTO expression varied significantly across different disease groups, emphasizing its potential as a reliable biomarker for diagnosing *S. aureus* infections.

FTO, located on chromosome 16q12.2, is primarily associated with obesity but has also been implicated in other diseases, including metabolic and inflammatory conditions. Previous studies have indicated that individuals with higher FTO expression tend to have a greater prevalence of *S. aureus* in their oral microbiota, suggesting a link between FTO and susceptibility to *S. aureus* infections, potentially through immune modulation and host-pathogen interactions (Huđek et al., 2017). This raises the possibility that FTO could play a critical role in the pathogenesis of *S. aureus*-induced osteomyelitis.

The role of FTO in inflammation is multifaceted and involves influencing m6A levels under stress conditions, such as hyperglycemia. Knocking out FTO in endothelial cells reduces inflammation and promotes cell migration and angiogenesis, suggesting a protective role against diabetic vascular damage via the FTO/TNIP1/NF-κB pathway. In ulcerative colitis, FTO downregulation exacerbates inflammation by altering sphingolipid metabolism, suggesting an m6A-dependent mechanism (Zhou et al., 2023). Overexpressing FTO in osteoarthritis models improves cartilage integrity and diminishes inflammation by modulating TLR4/MyD88/NF-κB signaling and NLRP3 inflammasome activity, which are relevant in myocardial ischemia/reperfusion injury. Moreover, FTO demethylase activity is crucial for innate immunity in cancer (Zheng et al., 2021). Combining FTO inhibition with a PD-1 blockade has the potential to enhance melanoma immunotherapy (Yang et al., 2019). In vascular parkinsonism, m6A RNA methylation can decrease the T helper cell count and mitigate vulnerable atherosclerotic plaques (Quan et al., 2021; Yang et al., 2020). These findings underscore the importance of FTO in regulating immune responses and inflammation in various contexts. Moreover, studies have shown that FTO expression is upregulated in hepatocellular carcinoma tumors, and targeting the FTO/m6A/GPNMB axis significantly inhibits tumor growth and metastasis while enhancing immune activation (Chen et al., 2024). In rheumatoid arthritis, FTO knockdown or inhibition significantly reduces the severity of arthritis (Li et al., 2024). In Parkinson’s disease, FTO knockout leads to a marked suppression of dopamine neuron death and restores the expression of tyrosine hydroxylase in the brains of PD mice (Geng et al., 2023). These findings underscore the potential of FTO as a therapeutic target for treating various diseases.

Our study, which utilized advanced machine learning and modeling, highlights the significant role of FTO in inflammatory diseases. We established an association between FTO expression and immune pathways in *S. aureus*-induced bone marrow inflammation. These pathways include IgA production within the intestinal immune network, PD-1 signaling, allograft rejection, and mycobacterial infection. Correlations with various immune cell activations underscore FTO’s regulatory impact on *S. aureus*-induced bone marrow inflammation. Our findings showed that METTL14 knockdown in bone marrow cells increased macrophage sensitivity to bacterial infections. LPS treatment increased SOCS1 m6A methylation and subsequently elevated SOCS1 levels by accelerating FTO mRNA degradation (Hu et al., 2022). Additionally, we noted marked differences in monocytes and macrophages between the disease and normal groups as well as within the m6A cluster grouping. In experiments with RAW 264.7 macrophages infected with *S. aureus*, increased MOI led to decreased FTO mRNA and protein expression levels. These findings support the notion that FTO plays a role in macrophage regulation during bone marrow inflammation. To further investigate the regulatory mechanism of FTO in macrophages during *S. aureus*-induced osteomyelitis, we utilized small interfering RNA (siRNA) technology to knock down FTO expression. Compared to the model group, we found that silencing FTO enhanced the inflammatory phenotype of macrophages, suggesting that FTO may modulate inflammation through the FOXO1/NFKB signaling pathway. This finding further supports FTO as a potential therapeutic target for modulating immune-inflammatory responses.

This study had a few limitations. First, because *S. aureus* is a live bacterium, it may be challenging to completely eradicate the bacteria effectively with the use of antibiotics during the handling of cells. This could potentially impact the results of the experiment, as the incomplete elimination of bacteria may lead to heterogeneity and uncertainty in experimental outcomes. Second, exploring differential gene expression within cells is valuable; however, it may not fully reflect the situation *in vivo*. Extracting proteins and mRNA from mouse models or human samples for detection may be more effective and reliable, as these samples closely resemble the real physiological environment. By studying animal models or clinical samples, a more accurate understanding of the changes in gene expression associated with, and mechanisms underlying, osteomyelitis could be achieved.

In conclusion, our study highlights the crucial role of the m6A RNA methylation regulator FTO in *S. aureus* infection-related osteomyelitis. It can serve as a potential biomarker for the diagnosis of *S. aureus* infection-related osteomyelitis and as a therapeutic target for modulating immune responses. This finding provides new insights into the relationship between FTO-mediated m6A RNA methylation and disease, laying the foundation for future research.

## Data Availability

The original contributions presented in the study are included in the article/Supplementary material, further inquiries can be directed to the corresponding author.
