# Supplementary figures and images for: The role of FTO in m6A RNA methylation and immune regulation in Staphylococcus aureus infection-related osteomyelitis
Source: Front Microbiol. 2025 Feb 6;16:1526475. doi: 10.3389/fmicb.2025.1526475 (PMC11839825; doi:10.3389/fmicb.2025.1526475)

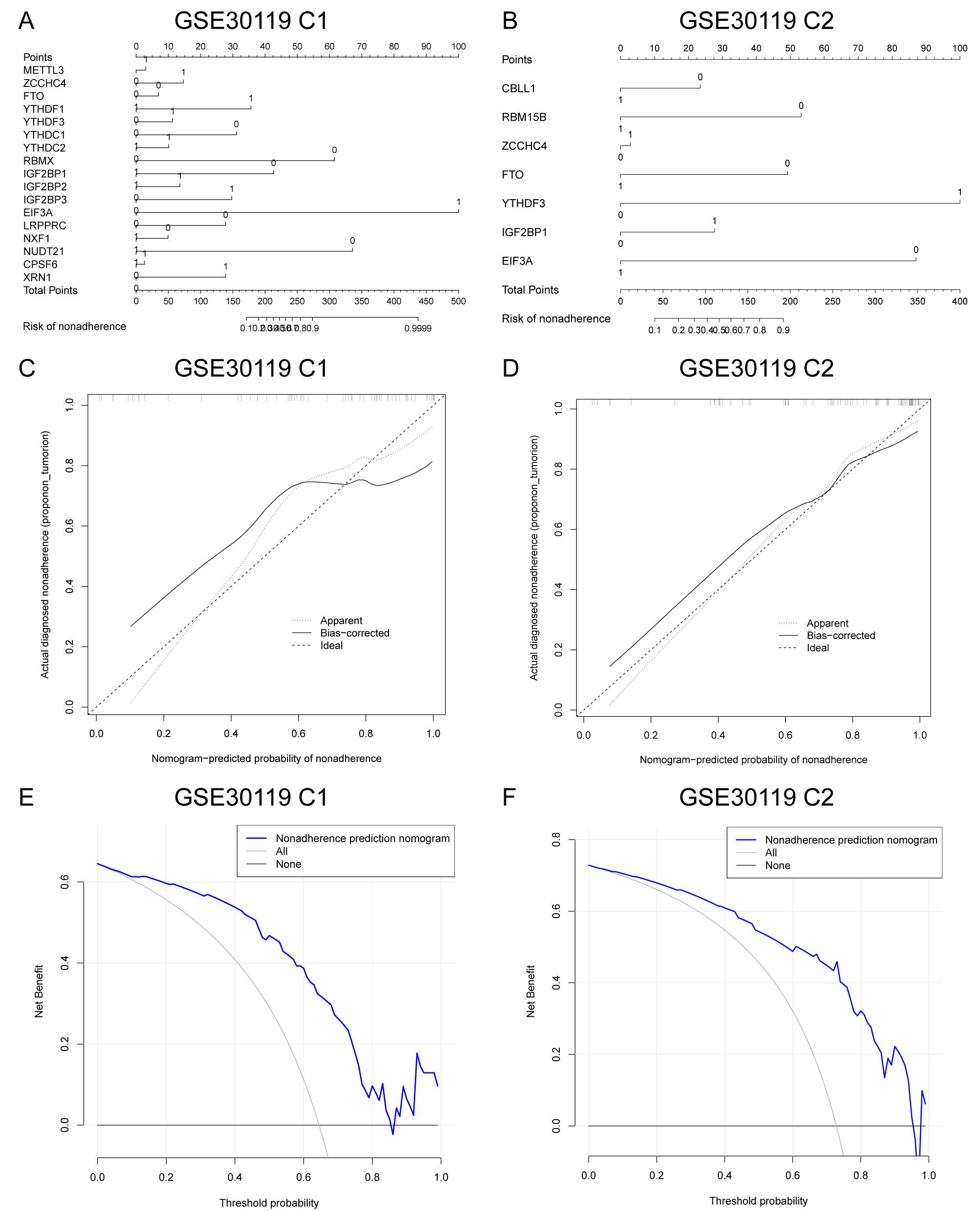

Supplement: Supplementary file 2 [file Image_2.TIF]

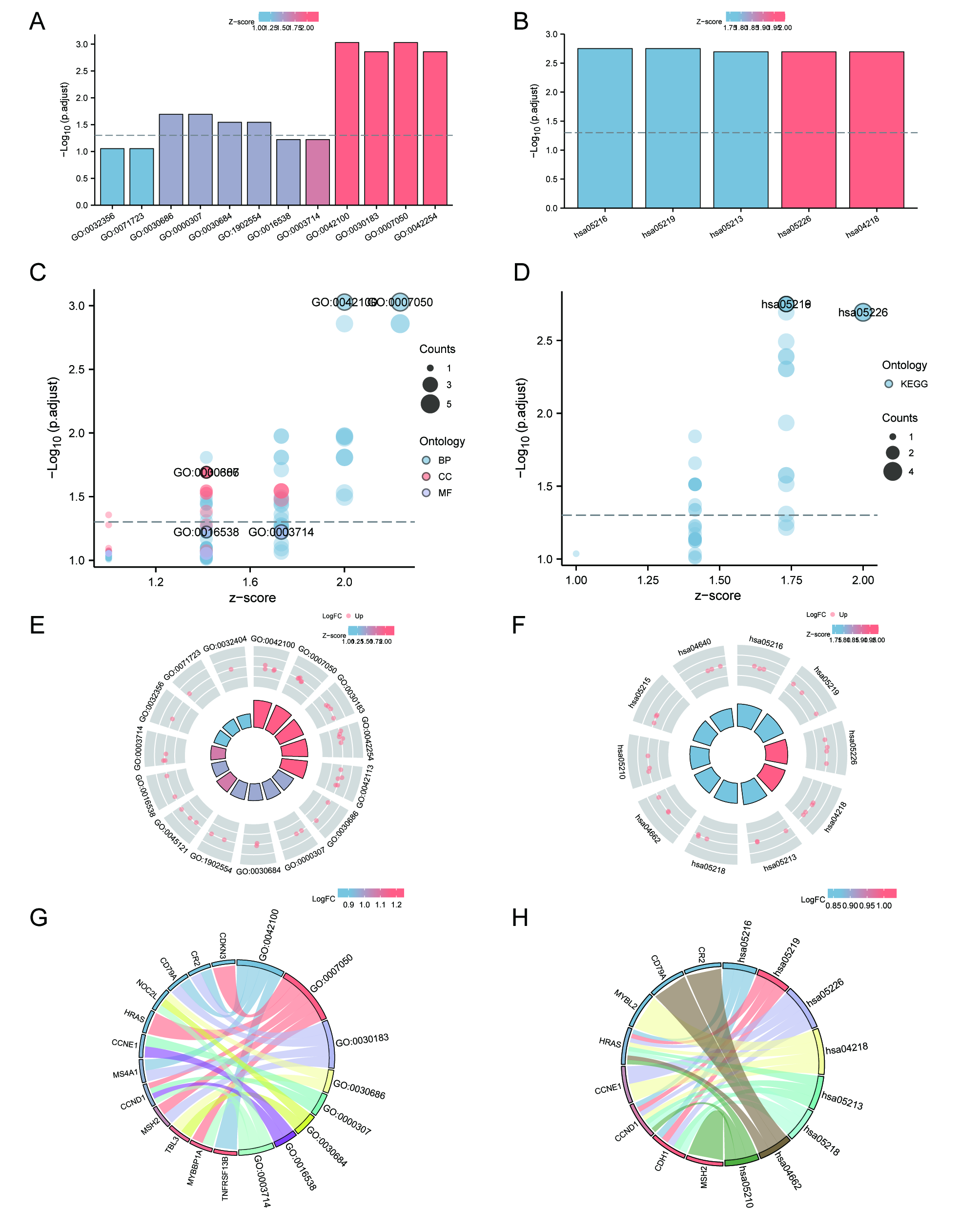

Supplement: Supplementary file 4 [file Image_4.TIF]

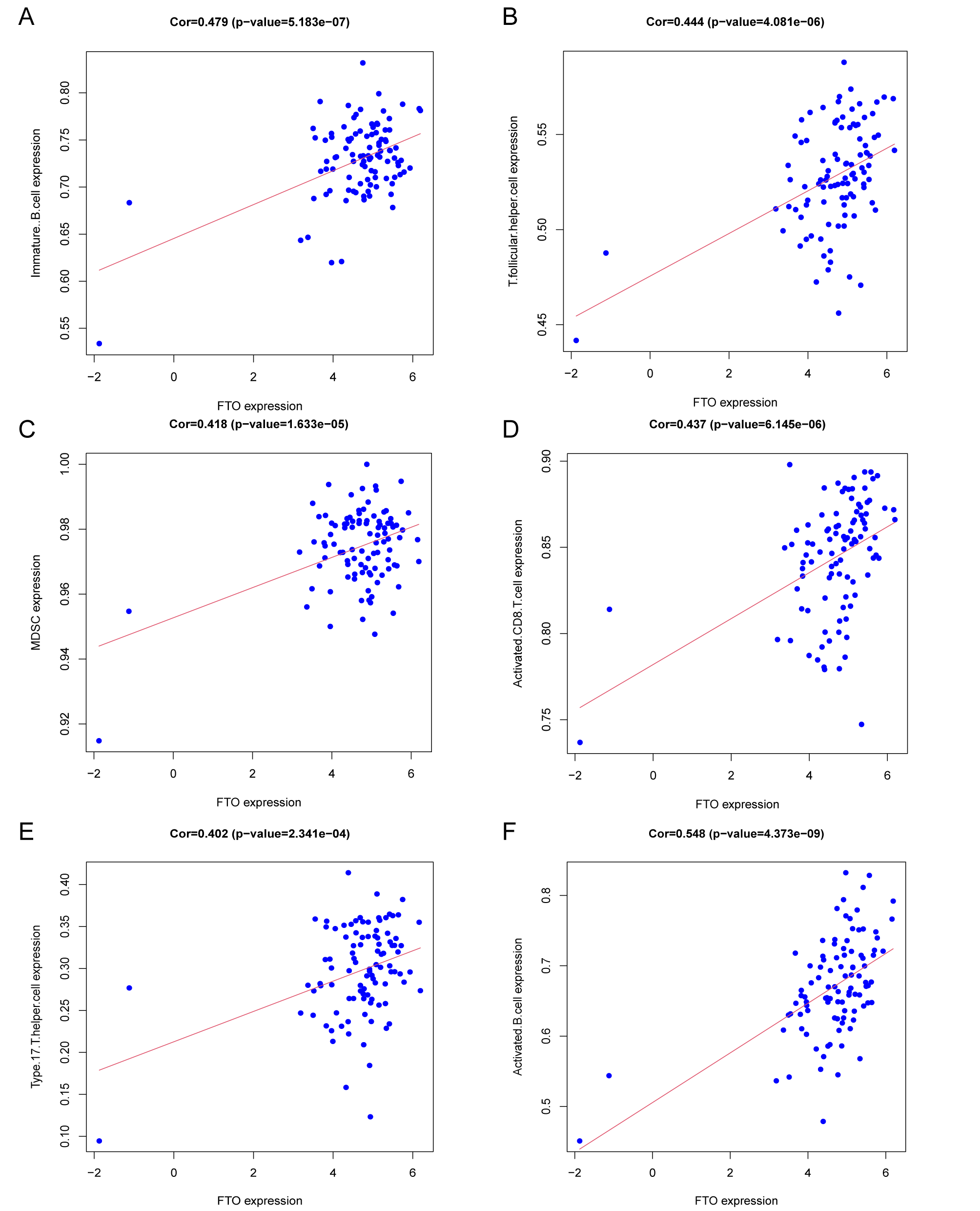

Supplement: Supplementary file 5 [file Image_5.TIF]

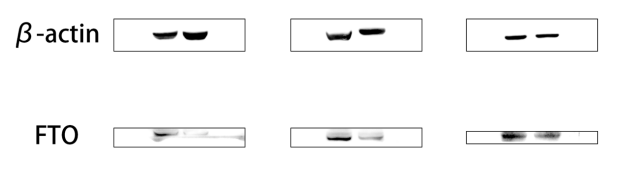

Supplement: Supplementary file 6 [file Image_6.PNG]
