# Supplementary material for: The role of FTO in m6A RNA methylation and immune regulation in Staphylococcus aureus infection-related osteomyelitis
Source: Front Microbiol. 2025 Feb 6;16:1526475. doi: 10.3389/fmicb.2025.1526475 (PMC11839825; doi:10.3389/fmicb.2025.1526475)
Supplement: Supplementary file 7 [file Table_1.DOCX]

Supplementary Material

# Supplementary table

**Supplementary Table 1**. Summary of biological process (BP), molecular function (MF), and cell component (CC) entries from GO enrichment analysis results of 19 co-expressed hub genes.

|  | **ID** | **Description** | **GeneRatio** | **BgRatio** | **p.adjust** | **q value** |
| --- | --- | --- | --- | --- | --- | --- |
| BP | GO:0042100 | B cell proliferation | 4/19 | 95/18670 | 9.37e-04 | 6.22e-04 |
| BP | GO:0007050 | Cell cycle arrest | 5/19 | 237/18670 | 9.37e-04 | 6.22e-04 |
| BP | GO:0030183 | B cell differentiation | 4/19 | 131/18670 | 0.001 | 9.19e-04 |
| BP | GO:0042254 | Ribosome biogenesis | 5/19 | 297/18670 | 0.001 | 9.19e-04 |
| BP | GO:0042113 | B cell activation | 5/19 | 310/18670 | 0.001 | 9.19e-04 |
| CC | GO:0030686 | 90S preribosome | 2/19 | 31/19717 | 0.020 | 0.013 |
| CC | GO:0000307 | Cyclin-dependent protein kinase holoenzyme complex | 2/19 | 42/19717 | 0.020 | 0.013 |
| CC | GO:0030684 | Preribosome | 2/19 | 76/19717 | 0.029 | 0.018 |
| CC | GO:1902554 | Serine/threonine protein kinase complex | 2/19 | 88/19717 | 0.029 | 0.018 |
| CC | GO:0045121 | Membrane raft | 3/19 | 315/19717 | 0.029 | 0.018 |
| MF | GO:0016538 | Cyclin-dependent protein serine/threonine kinase regulator activity | 2/18 | 49/17697 | 0.060 | 0.035 |
| MF | GO:0003714 | Transcription corepressor activity | 3/18 | 238/17697 | 0.060 | 0.035 |
| MF | GO:0032356 | Oxidized DNA binding | 1/18 | 10/17697 | 0.088 | 0.051 |
| MF | GO:0071723 | Lipopeptide binding | 1/18 | 10/17697 | 0.088 | 0.051 |
| MF | GO:0032404 | Mismatch repair complex binding | 1/18 | 11/17697 | 0.088 | 0.051 |

**Supplementary Table 2.** Summary of KEGG enrichment analysis results for 19 co-expressed hub genes.

| **Ontology** | **ID** | **Description** | **BgRatio** | **p value** | **p.adjust** | **q value** |
| --- | --- | --- | --- | --- | --- | --- |
| KEGG | hsa05216 | Thyroid cancer | 37/8076 | 2.45e−05 | 0.002 | 0.001 |
| KEGG | hsa05219 | Bladder cancer | 41/8076 | 3.35e−05 | 0.002 | 0.001 |
| KEGG | hsa05226 | Gastric cancer | 149/8076 | 6.99e−05 | 0.002 | 0.001 |
| KEGG | hsa04218 | Cellular senescence | 156/8076 | 8.36e−05 | 0.002 | 0.001 |
| KEGG | hsa05213 | Endometrial cancer | 58/8076 | 9.55e−05 | 0.002 | 0.001 |
| KEGG | hsa05218 | Melanoma | 72/8076 | 1.82e−04 | 0.003 | 0.002 |
| KEGG | hsa04662 | B cell receptor signaling pathway | 82/8076 | 2.68e−04 | 0.004 | 0.002 |
| KEGG | hsa05210 | Colorectal cancer | 86/8076 | 3.09e−04 | 0.004 | 0.002 |
| KEGG | hsa05215 | Prostate cancer | 97/8076 | 4.40e−04 | 0.005 | 0.003 |
| KEGG | hsa04640 | Hematopoietic cell lineage | 99/8076 | 4.67e−04 | 0.005 | 0.003 |

**Supplementary Table 3.** Staphylococcus aureus infection samples FTO express GSEA enrichment analysis need | NSE | result list.

| **ID** | **ES** | **NES** | **p value** | **q value** |
| --- | --- | --- | --- | --- |
| KEGG_INTESTINAL_IMMUNE_NETWORK_FOR_IGA_PRODUCTION | 0.721642 | 2.20886 | 0.001103 | 0.011623 |
| KEGG_ALLOGRAFT_REJECTION | 0.682395 | 2.015521 | 0.001121 | 0.011623 |
| KEGG_PRIMARY_IMMUNODEFICIENCY | 0.679027 | 1.99903 | 0.001121 | 0.011623 |
| REACTOME_PD_1_SIGNALING | 0.733168 | 1.992713 | 0.001189 | 0.011623 |
| BIOCARTA_TH1TH2_PATHWAY | 0.728048 | 1.978795 | 0.001189 | 0.011623 |
| BIOCARTA_CTLA4_PATHWAY | 0.715153 | 1.927325 | 0.001199 | 0.011623 |
| REACTOME_TRNA_PROCESSING | 0.584355 | 1.922247 | 0.001052 | 0.011623 |
| KEGG_ASTHMA | 0.673907 | 1.904362 | 0.001163 | 0.011623 |
| REACTOME_GENERATION_OF_SECOND_MESSENGER_MOLECULES | 0.662269 | 1.897139 | 0.001153 | 0.011623 |
| REACTOME_RRNA_PROCESSING | 0.541193 | 1.886609 | 0.001008 | 0.011623 |
| REACTOME_ANTIMICROBIAL_PEPTIDES | −0.61546 | −2.77118 | 0.016393 | 0.280684 |
| REACTOME_IRAK4_DEFICIENCY_TLR2_4_ | −0.76671 | −2.30653 | 0.004 | 0.141392 |
| REACTOME_BLOOD_GROUP_SYSTEMS_BIOSYNTHESIS | −0.64939 | −2.27776 | 0.00578 | 0.16483 |
| REACTOME_ERYTHROCYTES_TAKE_UP_CARBON_DIOXIDE_AND_RELEASE_OXYGEN | −0.73631 | −2.21509 | 0.004 | 0.141392 |
| REACTOME_INFECTION_WITH_MYCOBACTERIUM_TUBERCULOSIS | −0.57507 | −2.15502 | 0.006536 | 0.172579 |
| REACTOME_P130CAS_LINKAGE_TO_MAPK_SIGNALING_FOR_INTEGRINS | −0.63127 | −2.01179 | 0.00495 | 0.152808 |
| WP_SULFATION_BIOTRANSFORMATION_REACTION | −0.60405 | −1.99421 | 0.004926 | 0.152808 |
| REACTOME_GRB2_SOS_PROVIDES_LINKAGE_TO_MAPK_SIGNALING_FOR_INTEGRINS_ | −0.59776 | −1.905 | 0.00495 | 0.152808 |
| WP_SEROTONIN_TRANSPORTER_ACTIVITY | −0.61903 | −1.86227 | 0.012 | 0.238921 |
| KEGG_GLYCOSPHINGOLIPID_BIOSYNTHESIS_LACTO_AND_NEOLACTO_SERIES | −0.50685 | −1.84264 | 0.006536 | 0.172579 |
